# Development and Utilization of Tomato Microarrays for the *Solanaceae*

**DOI:** 10.1002/cfg.161

**Published:** 2002-04

**Authors:** Shanna Moore, Paxton Payton, Jim Giovannoni

**Affiliations:** 1 Boyce Thompson Institute for Plant Research Tower Road Cornell University campus Ithaca NY 14853 USA; 2 USDA-ARS Plant Soil and Nutrition Lab, Cornell University campus Ithaca NY 14853 USA


**Comparative and Functional Genomics**
*Comp Funct Genom* 2002; **3**: 164.
Published online 12 March 2002 in Wiley InterScience (www.interscience.wiley.com). **DOI:** 10.1002/cfg.161

**Conference Review**

### Development and utilization of tomato microarrays for the *Solanaceae*

Shanna Moore[1]*, Paxton Payton[1] and Jim Giovannoni[1,2]

[1] *Boyce Thompson Institute for Plant Research, Tower Road, Cornell University campus, Ithaca, NY, 14853, USA*
[2] *USDA-ARS, Plant, Soil, and Nutrition Lab, Cornell University campus, Ithaca, NY, 14853, USA*

**Correspondence to:*
*Boyce Thompson Institute for Plant Research, Tower Road, Cornell University campus, Ithaca, NY, 14853, USA. E-mail: slm47@cornell.edu*

Received: 24 January 2001
Accepted: 14 February 2002

The availability of an entire plant genome allows for many new insights into areas such as chromosome architecture, gene transfer from organelles, frequency and position of transposable elements, and evolutionary relationships. The task of sequencing an entire genome still remains formidable, however the relative ease and precision of sequencing and the efficiency of subsequent annotation and organization have allowed for the development of substantial EST databases for a growing number of plant species, including tomato. Although the available sequence information is substantial, it still falls short of encompassing all species of interest. Therefore, it is important to ask to what extent can the available sequence resources be exploited for functional or structural genomic analysis in additional species for which less information (and resources) are currently available.

Tomato (*Lycopersicon esculentum*) has long served as the model system for examining climacteric fruit ripening. Years of scientific investigation have resulted in substantial information and genetic resources for studying the biology of this agronomically important plant, including recently developed genomics tools. The advent of microarray technology now allows for the expansion from traditional platforms of forward and reverse genetics on a single gene basis to encompass the behavior of thousands of genes simultaneously. We have constructed a cDNA microarray for analysis of tomato fruit ripening and development. A fruit development gene statement profile is currently being created to establish a baseline of gene statement throughout development, with emphasis on ripening. We have also provided a description of a 9200 element array that will be available to the general public in March of 2002 (**http://bti.cornell.edu/CGEP/CGEP.html**). Additionally, we have demonstrated the potential use of tomato microarrays for genomics applications in other members of the *Solanaceae* family. Hybridization of leaf and fruit cDNA probes to tomato microarrays were employed to assess the potential utilization of this tool for gene statement profiling in tobacco, potato, petunia, pepper, and eggplant. Past work examining genome organization within the *Solanaceae* has shown that potato and tomato differ by only a few paracentric inversions, and although more rearrangements are evident between pepper and tomato, there is still substantial interspecies hybridization. This work, in addition to other studies in tomato, pepper, and potato have established the *Solanaceae* has having the best-characterized dicot comparative map. Our preliminary statement analysis using tomato microarrays and hybridization probes derived from widely related species confirm that tomato genomics tools will be broadly applicable across the *Solanaceae*. A majority of genes yielding a signal with tomato probes also yielded a signal with probes derived from potato, pepper, eggplant, tobacco and petunia. Gene statement data novel to each species was also detected. Global gene statement data, coupled with existing mapping data and corresponding proteome, metabolome and physiological variation among developmental stages or phenotypes will eventually provide specific targets for genetic manipulation in tomato, as well as other members of the family *Solanaceae*, yielding greater understanding of plant development and molecular tools for crop improvement.

Copyright © 2002 John Wiley & Sons, Ltd.

